# Renewed Concept of Mastoid Cavity Obliteration with the Use of Temporoparietal Fascial Flap Injected by Injectable Platelet-Rich Fibrin after Subtotal Petrosectomy for Cochlear Implant Patients

**DOI:** 10.3390/audiolres14020025

**Published:** 2024-03-01

**Authors:** Aleksander Zwierz, Krystyna Masna, Paweł Burduk, Stephan Hackenberg, Matthias Scheich

**Affiliations:** 1 Department of Otolaryngology, Phoniatrics and Audiology, Faculty of Health Sciences, Ludwik Rydygier Collegium Medicum, Nicolaus Copernicus University, 85-067 Bydgoszcz, Poland; krymasna@gmail.com (K.M.); pburduk@wp.pl (P.B.); 2 Department of Otorhinolaryngology, Plastic, Aesthetic and Reconstructive Head and Neck Surgery, University Hospital Würzburg, 97080 Würzburg, Germany; hackenberg_s@ukw.de (S.H.); scheich_m@ukw.de (M.S.)

**Keywords:** subtotal petrosectomy, cochlear implant, obliteration, temporoparietal facial flap, IPRF

## Abstract

**Background:** The subtotal petrosectomy procedure may be useful for cochlear implantation in selected patient groups. Although it is highly effective, complications can arise, which may have economic implications for the patient due to the high cost of the device. Therefore, several authors have attempted to identify the most effective concept for obliteration. **Methods:** We present a pilot descriptive study of application techniques for obliterating cavities after subtotal petrosectomy using a temporoparietal fascial flap (TPFF) modified with injectable platelet-rich fibrin (IPRF+) for three cochlear implant (CI) patients. **Results:** Our concept preserves important anatomical structures, such as the temporalis muscle, which covers the CI receiver–stimulator. Injection of IPRF+ also increases the available tissue volume for obliteration and enhances its anti-inflammatory and regenerative potential. **Conclusions:** To the best of our knowledge, the use of TPFF for filling the cavity has not been adopted for CI with SP and for blind sac closure. Our literature review and our experience with this small group of patients suggest that this procedure, when combined with IPRF+ injections, may reduce the risk of potential infection in the obliterated cavity, particularly when used with CI. This technique is applicable only in cases when the surgeons are convinced that the middle ear cavity is purged of cholesteatoma.

## 1. Introduction

Cochlear implantation via the trans-mastoid approach through the facial recess has become the standard procedure for treating patients with profound sensorineural hearing loss. However, there is a dispute among researchers regarding the management of chronic ear discharge with or without cholesteatoma. The debate centers on whether to eradicate the underlying pathology, cover the electrode, and leave the cavity open, or to perform other challenging approaches, such as via the middle cranial fossa or through subtotal petrosectomy (SP) [1,2]. Szymański, in particular, reviewed the results of “covering techniques” performed by Olgun and Roehm. These techniques involved wrapping the electrode cable into the patient’s dense tissues and resulted in a major complication rate of 14–19%, mainly due to electrode cable disruption. Therefore, he strongly discouraged the use of these techniques [2]. Moreover, he did not recommend the use of “bypass techniques” and electrode insertion from the diseased middle ear [2]. The only recommended technique was subtotal petrosectomy performed with external auditory canal (EAC) closure, since it isolates and seals the surgical cavity from the external environment, consequently leading to a lower risk of infection and CSF leak prevention, as well as providing a good exposure of the round window [3].

Bone dust, abdominal fat, cartilage, muscle, local flaps, and hydroxyapatite, among other things, have been used to fill the cavity in the SP procedure [4]. Since all these obliterative materials vary in the scope of available volume, vascularity, resorption rate, scarring, and damage to the donor site, identifying their advantages and disadvantages may help determine their indication of use.

A one-stage cochlear implant (CI) implantation within an infected middle ear cavity, affected by a chronic inflammatory process, necessitates a scrupulous cleansing of the inflammatory cavity. Additionally, it requires the utilization of tissues endowed with a robust blood supply, preferably local flaps, to effectively obliterate the cavity. In this context, the temporoparietal fascial flap (TPFF) emerges as an optimal choice. Positioned in close proximity to the ear cavity, the TPFF boasts a vascular pedicle arrangement conducive to rotational adjustments without compromising vascular flow. Notably, the TPFF is distinguished by its status as the thinnest pedunculated flap in humans, and its harvesting is devoid of any visible tissue defects [5].

To enhance the regenerative potential of this procedure, we incorporated injectable platelet-rich fibrin (IPRF+), an agent manifesting significant clinical utility. The advantageous impact of IPRF+ on expediting wound healing, mitigating postoperative hematoma, edema, and pain, and even addressing connective tissue defects is substantiated by a myriad of studies across diverse medical domains [6]. Its widespread application spans dermatology, where it is utilized for skin regeneration and the treatment of various wounds, exerting influence on angiogenesis and facilitating skin recovery post-irradiation [7,8,9,10]. The angiogenic properties of IPRF extend its utility to enhancing the survival of free skin grafts and fostering muscle regeneration [11,12]. IPRF has found extensive application in dentistry, with numerous studies attesting to its superiority in treating gingival recession, interdental deficiencies, and promoting healing after flap harvesting [13,14]. Furthermore, the antimicrobial activity of IPRF, as affirmed by Straub’s study, adds another layer of efficacy to its multifaceted utility [15].

IPRF+ administration, being a time-efficient and cost-effective procedure, holds the potential to significantly influence treatment outcomes. Thus, in the pursuit of augmenting the effectiveness of postoperative healing, achieving the requisite tissue volume to fill the entire postoperative cavity with a flap, and preempting infections, we judiciously employed a temporoparietal fascial flap injected with IPRF+.

## 2. Materials and Methods

Herein, we present our concept of cochlear implantation in cases of SP procedure with cavity obliteration using the temporoparietal fascial flap (TPFF) (Byrd, 1980) augmented with injectable platelet-rich fibrin (IPRF+). The technique was used in three patients in the last 2 years. The affirmative feedback received thus far urged us to present a pilot descriptive study of implementation of this surgical treatment.

**Figure 1:**
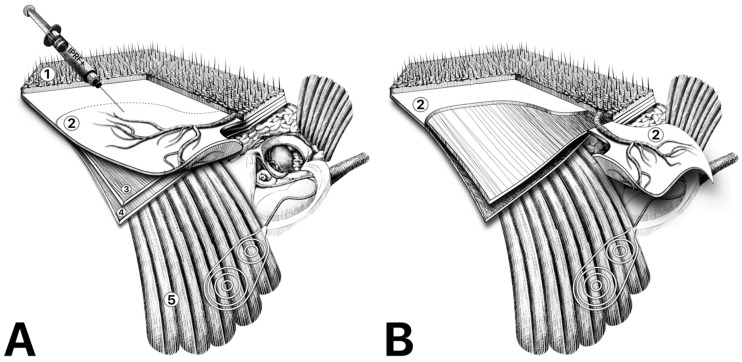
(**A**) Flap preparation and IPRF+ injection. Anatomic layers of the temporoparietal region: 1—skin and subcutaneous tissue, 2—temporoparietal fascia, 3—loose areolar tissue, 4—temporalis muscle fascia, 5—temporalis muscle. (**B**) Temporoparietal fascial flap rotated into subtotal petrosectomy cavity.

Initially, receiver–stimulator placement was planned on the skin. A wide retroauricular incision was then made, and a continued vertical incision was traced from the root of the helix towards the superior temporal line. The incision was lined up to the superior margin of the planned TPFF, based on both the parietal and frontal branches of the superficial temporal artery (STA), as identified by Doppler imaging or by palpation. Usually, the STA is located 2 cm superiorly and anteriorly from the EAC, running along with one or two veins located above the artery. The skin incision was then carried down to the TPFF and the mastoid periosteum. After identification of the EAC posterior wall, a C-shaped periosteal flap was created 5 mm posterior from the EAC, and a posteriorly horizontal incision along the linea temporalis was performed. Following this, the periosteal flaps were prepared, and a subperiosteal pocket for the receiver–stimulator was created. The mastoid and middle ear cavity were then cleared from the pathological tissue by mastoidectomy with complete exenteration of pneumatic cells and identification of the vertical facial nerve segment. Afterwards, the facial recess was opened, and if present, the incudostapedial joint was disconnected. The skin of the posterior and anterior external auditory bony canal was horizontally incised and lifted, and the posterior canal wall with the tympanic membrane, malleus, and incus were removed. After removing the underlying pathology and all of the middle ear mucosa, the Eustachian tube was obliterated with the free muscle tissue, and the skin of the EAC was everted in its ostium and then sutured with 3-0 absorbable sutures, supported by the inverted cartilage of the EAC. Following this, the TPFF was prepared. The anterior and posterior scalp flaps were elevated through meticulous sharp dissection in the subdermal plane, taking care to continue the dissection slightly below the hair follicles to leave a thin layer of subcutaneous fibrofatty tissue to prevent local alopecia. The STA was then identified 0.5–1 cm anterior to the root of the helix, and the dissection was continued peripherally along the vessel to prepare the entire area of the flap. Afterwards, the flap was raised caudally from the loose areolar tissue and was tailored to enter the open cavity in the anterior–superior direction (Figure 1). Subsequently, the round window niche was identified, the round window was exposed, the receiver–stimulator was fixed, the round window was opened, the electrode was inserted into the cochlea, and then the opening was sealed. Moreover, prior to insertion, 10 mL of blood was collected in each of the six sterile IPRF+ 13 mL tubes, and underwent centrifugation at 700 rpm for 5 min (Duo Quattro PRF centrifuge). Following this, the superficial part of the liquid was taken using a syringe, which gave approximately 8 mL of IPRF+. The TPFF was then evenly injected with IPRF+ and rotated so that IPRF+ filled the entire cavity. After approximately 20 min of stiffness, the temporalis muscle, periosteal flap, subcutaneous tissue, and skin were all sutured. Lastly, pressure dressing was applied for 48 h.

## 3. Results

### 3.1. Case 1

**Figure 2:**
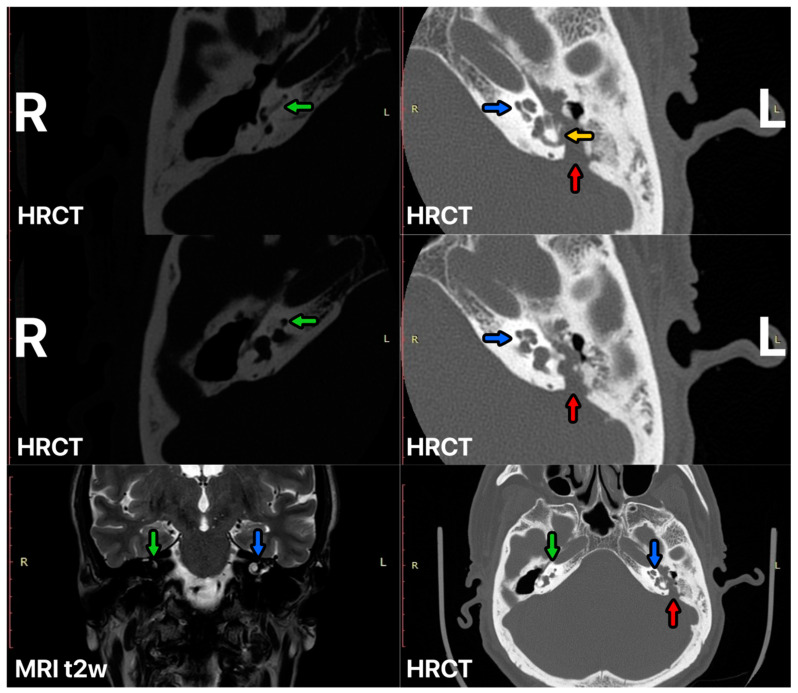
HRCT and MRI of the ear before SP surgery. Right cochlear ossification—green arrow, left cochlear—blue arrow, posterior fossa destruction by cholesteatoma—red arrow, lateral semicircular canal destruction—yellow arrow.

**Figure 3:**
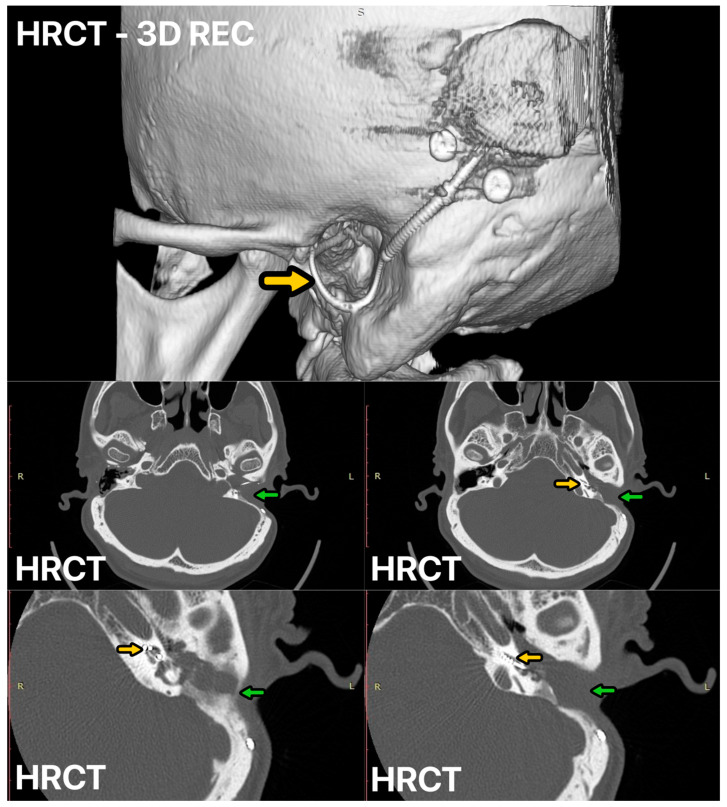
HRCT of the left ear 12 months after surgery, CI electrode—yellow arrow, mastoid cavity obliterated with temporoparietal fascial flap (TPFF) injected with injectable platelet-rich fibrin (IPRF+)—green arrow.

**Figure 4:**
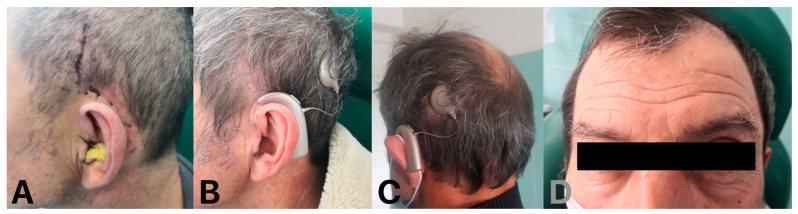
(**A**) Patient #1 after 3 weeks, (**B**,**C**) after 12 months, (**D**) full symmetry of the head.

A 59-year-old man was admitted to our clinic with a history of bilateral chronic otitis media with cholesteatoma. His right ear was operated on many years ago and a canal wall down procedure was performed. The inflammation caused deafness in the right ear with cochlear ossification (Figure 2). After many years, left-side cholesteatoma gradually destroyed the left ear, impaired the hearing, and finally caused complete deafness. We performed left-side SP with mastoid cavity obliteration with a temporoparietal fascial flap injected by injectable platelet-rich fibrin and a one-stage CI procedure. A full insertion with the cochlear implant placed in the scala tympani was achieved (Figure 3). After rehabilitation, the patient developed audiological performance of open-set word testing with the range of 90%. We present very good aesthetic results of the obliteration obtained by the usage of TPFF injected with IPRF+ (Figure 4). We did not observe any complications within period of 15 months.

### 3.2. Case 2

**Figure 5:**
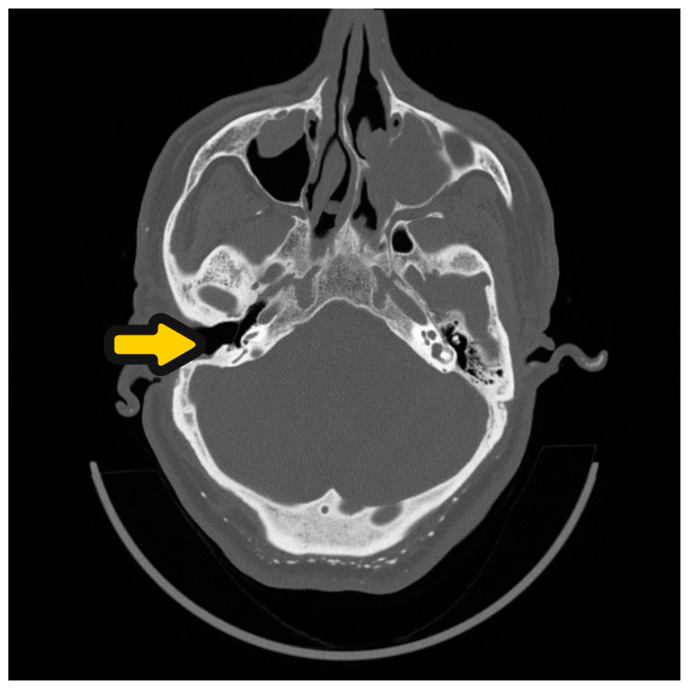
HRCT of the ear before SP and CI procedure. Open right-ear cavity after first ear surgery—yellow arrow.

**Figure 6:**
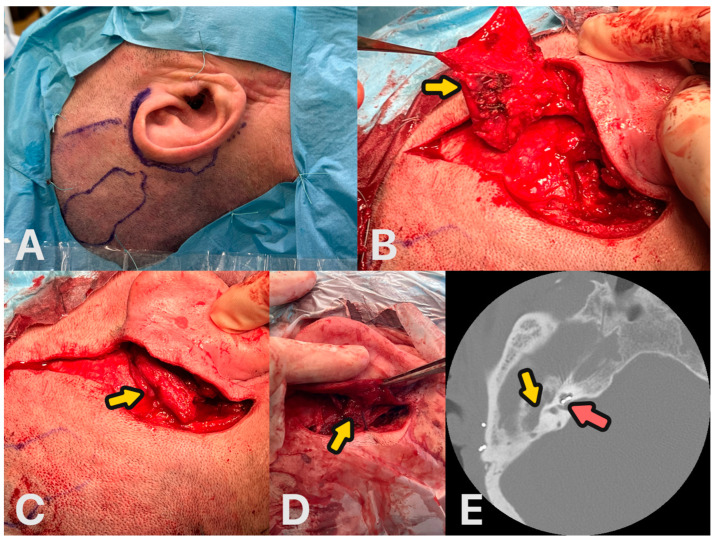
Surgical steps of the obliteration. (**A**) Planning of the surgery. (**B**) Harvesting of the temporoparietal fascial flap—yellow arrow. (**C**) Temporoparietal fascial flap rotated to subtotal petrosectomy cavity—yellow arrow. (**D**) Ear closing—yellow arrow. (**E**) Control CT scan 12 months after surgery, obliterated cavity—yellow arrow. CI electrode in the cochlea—red arrow.

A 68-year-old man, who had experienced bilateral deafness, was admitted to the clinic. Several years prior, he had undergone surgery on his right ear due to chronic otitis media with cholesteatoma. The procedure involved a canal wall down ear surgery and tympanic membrane reconstruction; however, ossiculoplasty was not performed during this operation (Figure 5). At that time, the patient had already developed right-sided conductive hearing loss in the right ear, which was effectively managed with a hearing aid. He experienced progressive bilateral sensorineural hearing loss, rendering bilateral hearing aids ineffective for a period of 4 years. There was stated discharge from the right ear but without cholesteatoma recurrence. As a result, the patient became a candidate for right-sided cochlear implantation. A one-stage procedure was executed, involving subtotal petrosectomy and cochlear implantation. A temporoparietal fascial flap obliteration with augmentation using injectable platelet-rich fibrin was also performed during the procedure (Figure 6). The postoperative course proceeded without complications, characterized by rapid tissue healing and the absence of fistulas or wound-related inflammation.

### 3.3. Case 3

**Figure 7:**
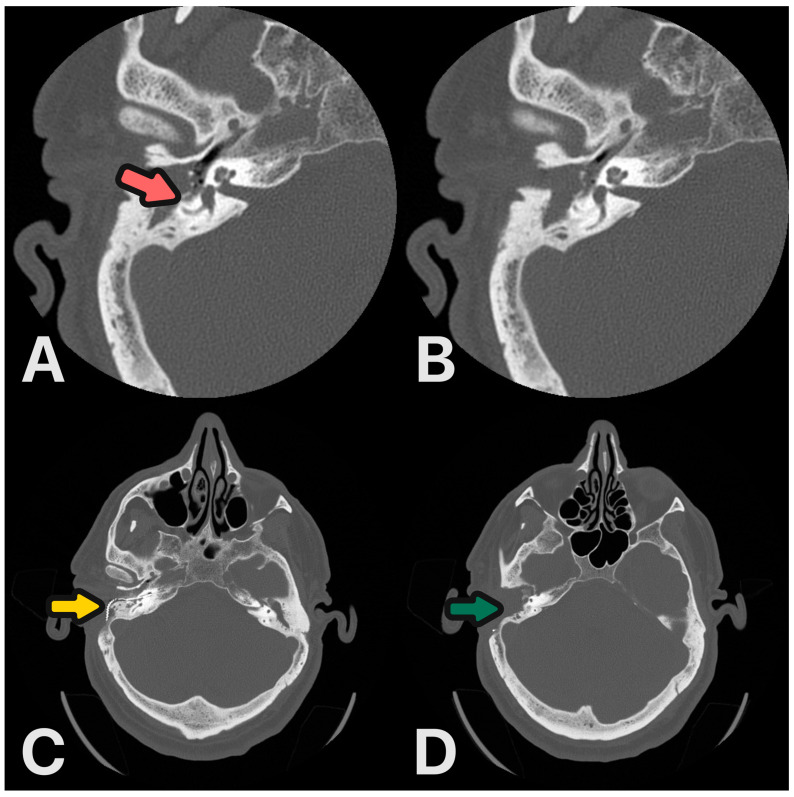
(**A**,**B**) HRCT scans before surgery: destruction of the lateral semicircular canal and posterior canal wall—magenta arrow. (**C**,**D**) Control HRCT scans 6 months after procedure: CI electrode—yellow arrow, ear obliterated—green arrow.

A 69-year-old man sought admission to the clinic due to sudden right-sided deafness and dizziness that had persisted for two months. Computed tomography revealed the presence of an extensive cholesteatoma in the tympanic cavity and mastoid region, with associated destruction of the lateral semicircular canal, categorizing it as a type III labyrinth fistula according to the Dornhoffer and Milewski classification (Figure 7). Given the potential risk of cochlear fibrosis subsequent to cholesteatoma removal from the lateral semicircular canal, a one-stage surgical intervention was planned. This consisted of a lateral petrosectomy with obliteration using a temporoparietal fascial flap (TPFF) augmented with 2 mL of injectable platelet-rich fibrin (IPRF+), along with simultaneous cochlear implantation (Figure 7). In this case as well, healing progressed properly, and the patient rapidly gained the ability to utilize the cochlear implant.

It is particularly important to precisely remove the pathology in order to minimize the risk of cholesteatoma recurrence. In each of the described cases, we were convinced during the surgery that the cholesteatoma was completely removed; if this was not the case, we were always prepared to put a dummy electrode and perform the procedure in two stages. All patients are still under our medical supervision.

## 4. Discussion

The idea of subtotal petrosectomy for pathological ear treatment has been established for the past few decades. It was first described by Mosher in 1911; its concept was then retaken and modified by Rambo in 1958, was finally named by Ugo Fisch in 1965, and was further modified by other famous and experienced ear surgeons, such as Gacek and Palva [4]. Notably, the procedure described by Rambo did not involve closure of the external auditory canal, since he explained that its closure minimizes ear discharge. With these developments over the years, SP has become an effective method for eliminating recurrent ear infections, removing large tumors, obliterating the middle ear and mastoid, and eliminating the possibility of intracranial spread from infections, always with the possibility of CI implantations in difficult cases [4].

**Figure 8:**
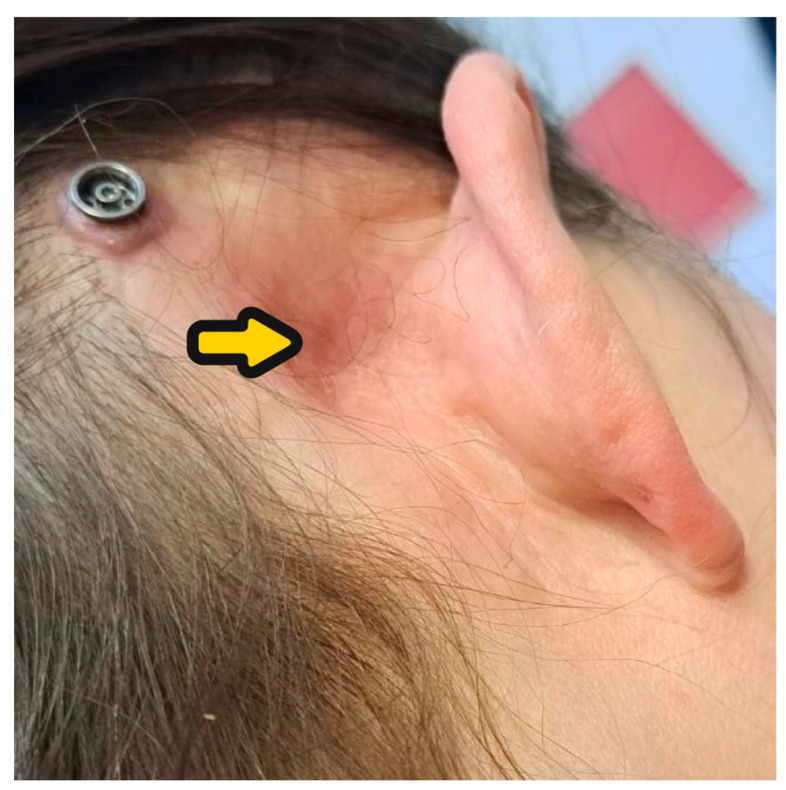
Patient 12 months after SP with external auditory canal closure and fat obliteration. Yellow arrow shows a depression caused by absorption of fat.

Regarding the filling used for SP, Rambo reported the use of the temporalis muscle for obliteration, while in 1976, Gacek presented the use of abdominal fat and an inferiorly based muscle pedicle [4]. Brizgalis suggested that the temporalis muscle flaps in SP need to be small enough to allow overfilling of the large cavity to compensate for its gradual postoperative shrinkage [1]. However, Bartells has reported complaints of severe pain above the ear and cases of necrosis following temporalis muscle flap creation [16]. On the other hand, free tissue grafts, such as abdominal fat, cartilage or bony palate, lack vascularity and are subject to resorption [17]. Particularly, Yung noted postoperative infections and fat necrosis in a number of patients obliterated with abdominal fat [18], which was consistent with our findings and that of other studies. Moreover, Bernardeschi reported that 8% of minor complications at the donor site were abdominal hematomas [19]. Giulia D’Angelo similarly presented one case of seroma and one case of hematoma in 32 patients who underwent SP with abdominal fat grafting [20]. In these cases, other sources of free grafts are recommended as an alternative, such as fat or temporalis muscle. In 1996, Chenay introduced an interesting concept of using the pedicled TPFF for cavity obliteration in the SP procedure [21]. TPFF has been found to have a beneficial effect on the healing and acceptance of free skin grafts for mastoid obliteration due to its resistance to bending and compression as a result of the large diameter of the pedicle vessels [17,22]. Therefore, based on the results of Chenay and Yung, the author of this article used TPFF for obliteration after the SP procedure [18,21]. However, problems regarding the flap’s maximal evaluable volume for cavity obliteration and postoperative muscle shrinkage were noted. The TPFF, in particular, has a flat surface (150 cm^2^) and is the thinnest in the human body pedicle flap (2 mm), only providing a volume of 30 mL, which may be a limitation for its use. For this reason, we used up to 8 mL IPRF+ injections to increase the flap volume, given its high vascularity and regenerative ability [23]. IPRF+ is a second-generation, fully autologous, blood-derived biomaterial with a three-dimensional fibrin meshwork that is widely used in dentistry, dermatology, and orthopaedics [24]. Furthermore, IPRF+ preparation is easy and requires a basic instrument set without biochemical blood handling [25]. IPRF+ is widely used due to its high regenerative potential from its plates and lymphocytes, which deliver growth factors. Moreover, it consists of type-1 collagen, making it useful in various surgical conditions [24,26], and its anti-inflammatory role in wound healing is also essential for postsurgical recovery [27]. In summary, it is important to note the advantages and disadvantages of the commonly used SP obliteration materials. Although fat has a high volume, it has poor vascularization and has a tendency for inflammation and absorption (Figure 8). On the other hand, the temporalis muscle flap has a lower volume availability that is needed for rotation, which creates a situation of incomplete coverage of the receiver–stimulator. The flap also has a tendency to shrink, but it has good vascularity, providing efficient anti-inflammatory resistance. Lastly, TPFF injected with IPRF+ demonstrates all the advantages of these two reconstructive materials, while providing the best cosmetic effect.

The first cases of SP and cochlear implantation were reported by Bendet and Issing in 1998 [28,29]. SP and CI placements are especially useful in the treatment of chronic ear disease with or without cholesteatoma, cerebrospinal fluid (CSF) leakage, middle and inner ear malformations, cochlear obliteration, and ossifications requiring partial drilling in cases of difficult insertion [2,4,30]. Szymański, in accordance with Fisch’s teaching techniques, proposed two variants of surgery, depending on whether the ear has been previously operated or not [2]. In unoperated patients, the gap was closed using fat covered with free fascia graft from the temporalis muscle and the musculoperiosteal flap. For previously operated patients, fat coverage using the temporalis muscle rotated inferiorly was recommended [2]. Despite the good results in the second patient group, the receiver–stimulator was not completely covered by the temporalis muscle, which may have possibly impacted the extrusion of the receiver–stimulator. Moreover, although Polo reported a low rate of major complications (6.37%) in 110 cases of SP and CI insertion performed by the Grupo Otologico in Piazenza [3], a reduction of this incidence should be attempted, both for the patient’s health and for the economic burden due to the cost of the device. Specifically, the major complications of SP and CI included fat infection, sac closure breakdown, entrapped cholesteatoma, and extrusion of the CI electrode or the receiver–stimulator [31]. If these procedures are performed with care, external canal skin closure would prevent its breakdown. This is equally important in second-layer closure with cartilage, which was performed using the Grupo Otologico principles, resulting to no cases of blind sac breakdown [32,33]. In contrast, Leung reported one case of blind sac breakdown among 17 patients who underwent Melbourne SP procedures, emphasizing again the importance of performing with care [30]. Additionally, meticulous surgical techniques have reduced the risk of cholesteatoma recurrence in SP to 1.5% [4], which is less in comparison to that of the canal wall down procedure (1.5–15%), as described by Prasad. In Leung’s and D’Angelo’s SP patients with CI, cholesteatoma recurrence was not observed; however, the follow-up periods were too short to reach a definitive conclusion in both studies [20,30]. In contrast to these studies, Altunia reported an 8% complication rate of fat inflammation that required CI removal [31]. Given these reported complications, the proposed obliteration with well-vascularized TPFF and IPRF+ injections should reduce the incidence of inflammation and accelerate the healing process. Furthermore, device migration and exposure may occur, moving or rotating the temporalis muscle flap and resulting in complications. This was seen in Leung, who strongly preferred temporalis muscle flap obliteration, reporting one case of potential exposure among the 17 reported operations [30]. Interestingly, Hernandez proposed the application of the other concepts in three of his cases [33]. In his attempt to avoid all complications related to obliteration, he proposed a controversial technique of SP and CI with ear canal and Eustachian tube obliteration, but without mastoid obliteration [34].

The use of the presented technique in all of the above cases resulted in favorable outcomes, with complete ear obliteration achieved without postoperative infections or wound breakdowns. As a result, middle-ear pathology was successfully resolved, cochlear implants were securely positioned, hearing was restored, there was no evidence of head asymmetry, and a satisfactory cosmetic outcome was achieved. During the different periods of treated patient observation, from 9 to 15 months, we did not observe the occurrence of severe symptoms such as headache and local soft tissue inflammation, which may indicate a possible recurrence of cholesteatoma. We scheduled a follow-up MRI 1.5 T (Non-Echo Planar Diffusion-Weighted Imaging) 1.5 years after the procedure, bearing in mind that MRI scans are sometimes deteriorated by artifacts and had limited applicability on the CI side [35].

## 5. Conclusions

To the best of our knowledge, the concept of using TPFF to fill the cavity has not been adopted for CI with SP and for blind sac closure. The combination of this procedure with IPRF+ injections may reduce the risk of potential infection in the obliterated cavity, which is especially important when the procedure is performed with CI. Our concept preserves important anatomical structures such as the temporalis muscle, which covers the CI receiver–stimulator and may prevent extrusion complications. Injection of IPRF+ also seems to increase the volume of tissue available for obliteration and enhances its anti-inflammatory and regenerative potential. Compared to the use of fat, TPFF with IPRF+ injections may reduce the incidence of necrosis, tissue absorption, and poor cosmetic effects, such as retro auricular holes and abdominal scars. Moreover, raising of the TPFF, the thinnest pedicled flap, does not change the shape of the head (Figure 4).

In addition, obliteration with TPFF and IPRF+ injections may still be used in patients who had previously undergone ear surgery with other local muscular obliteration, including Palva flaps and superiorly or inferiorly based muscular pedicle flaps [21,23].

It should be emphasised that this technique is applicable only in the case when the surgeons are convinced that middle ear cavity is purged of cholesteatoma.

## Data Availability

All data are available for request.
